# Eco-destination loyalty: Role of perceived value and experience in framing destination attachment and equity with moderating role of destination memory

**DOI:** 10.3389/fpsyg.2022.908798

**Published:** 2022-08-23

**Authors:** M. Mengkebayaer, Muhammad Asim Nawaz, Muhammad Umar Sajid

**Affiliations:** ^1^Tourism College, Inner Mongolia Normal University, Hohhot, China; ^2^School of Management, University of Science and Technology of China, Hefei, China; ^3^Lyallpur Business School, Government College University, Faisalabad, Pakistan

**Keywords:** ecotourism, perceived value, experience, destination attachment, destination equity, memory

## Abstract

This research article aims to evaluate the characteristics of ecotourism destination loyalty in light of destination attachment, destination equity framed by perceived value, and tourist experience. Thus, the attributes of ecotourism destination branding in formulating tourist loyalty are examined. The study is of significant importance for developing economies having natural tourist destinations. A total of 358 questionnaires were filled through wjx, and a SmartPLS-based structural equation modeling tool was used to analyze the data obtained from eco-tourists. The software is essential for complex structural models, including multiple indicators, and relationships. The empirical results exhibit that perceived value and tourist experience significantly contribute to destination loyalty and equity, eventually influencing tourist destination loyalty. Moreover, destination memory moderates the relationship between destination attachment, destination equity, and destination loyalty. Further, destination attachment and destination equity mediate the relationship between the perceived value, experience, and destination loyalty. Additionally, the study extends the tourist consumption theory to the ecotourism literature. Besides the theoretical contribution, the study makes a practical contribution to practitioners. For instance, perceived value is a prime contributor to tourist destination loyalty. In perceived value, the most important factor is good value for money. Such practical contribution will provide a pathway to the strategic formation of business.

## Introduction

Ecotourism is an emerging format in the tourism industry. The ecotourism industry worldwide has shown enormous growth and has made $181.1 billion in 2019 (Statista, 2021). The segment is forecast to reach $333.8 billion by 2027, with an expected CAGR of 14.3% (Statista, 2021). United Nations declared 2002 the “international year of ecotourism” to encourage sustainable tourism development (Karst and Nepal, 2021). Ecotourism, as a tourism segment, is an industry that contributes to 10% of the world GDP, 7% of world tourism, and generates 10% of employment (World Health Organization, 2019). Furthermore, ecotourism accounts for one-third of the tourism crowd (World Health Organization, 2019). China is investing heavily in the ecotourism sector, as the country is ranked second in the world for travel and tourism contribution to the GDP and first in tourism-based employment generation (Statista, 2021). Directly or indirectly, the tourism industry contributes ~10% of the country's GDP (Statista, 2021). Ecotourism (ET) has gained popularity in the Chinese environment since the 1990s. ET helps construct a strong bond between nature and humans with the prime objective of sustainable business development. Eco-tourist destinations generate socio-economic benefits for the local and rural populations regarding employment and business. Ecotourism is helping to reduce the unemployment that causes the population shift from rural to urban areas (Ramírez and Santana, 2019).

This causes fierce competition between the business organizers to give exceptional services to visiting tourists. Attracting and retaining existing customers is the backbone of any business and a vital ingredient of survival and development in the tourism industry. Perceived value (PV) and experience (EX) are the two essential contributors to behavioral intentions of attraction (Schenk et al., 2008; Ha and (Shawn) Jang, 2010). Destination attachment (DA) is one of the fundamental characteristics of revisit traveling behavior, which makes it an essential indicator of the tourist decision-making process regarding destination selection (Cifci, 2021). Moreover, destination equity (DE) derived from brand equity is a differentiating factor in tourist knowledge, influencing tourist preferences and behavior toward the destination (Kumail et al., 2021). Moreover, the tourist perception of value and prior experience contributes enormously to the destination attachment and equity, eventually leading to the revisit intention (Chang and Huang, 2014). The constructs of value perception and experience have led to several studies that explore the tourist experience and their revisit intention. This study considers the perceived value and experience as antecedents of destination attachment. It enhances destination equity simultaneously, and they eventually encourage tourists to revisit eco-destinations. Attachment and equity have led to numerous branding exploration icons and are recognized as essential factors in framing the consumer repurchase intention (Spry et al., 2011; Dwivedi et al., 2019), such as social media (Dwivedi et al., 2019), celebrity endorsement (Spry et al., 2011), and brand awareness (Chandon, 2003). But few studies have discussed destination attachment and destination equity in the ecotourism context. Moreover, this study offers the moderating role of destination memory. Destination memory is the primary factor in tourist intention studies (El Haj and Miller, 2017). In this study, we consider the memories in the context of the prior visit's unforgettable events. For tourists, memories have a pivotal role in motivating revisit intention (Kim, 2020).

## Literature review

### Ecotourism destination loyalty

In consumer behavior, loyalty means a commitment to a specific product (So et al., 2013; Hew et al., 2016; Chang, 2021). So, in a tourism context, destination loyalty means tourist commitment to a particular destination (Lee and Xue, 2020; Mirzaalian and Halpenny, 2021). Social scientists have two solid reasons for continuous exploration of destination loyalty and allied behavioral response. First, destination loyalty helps to generate economic activity for the local population through the general value perception and experiences associated with the natural sites (Kuo and Feng, 2013; El-Adly, 2019). Second, novelty-seeking influences tourist traveling motivation that guides the decision-making process (Ramírez and Santana, 2019). This means destination loyalty is more complex than customer loyalty, requiring more effort to develop understanding.

Social scientists continuously explore the characteristics that can influence tourist loyalty, considering the practical implication of destination loyalty. Tourist loyalty studies can be summarized as motivation factors (Suhartanto et al., 2020a), demographic characteristics (Stojanovic et al., 2017), past experiences (Chang et al., 2014), destination image (Lee and Xue, 2020), service quality (Alexandris et al., 2006), perceived quality (Shahijan et al., 2018), satisfaction (Quynh et al., 2021), and novelty (Chang et al., 2014). Based on these antecedents, the researchers have developed numerous theoretical frameworks to understand the formation process of tourist loyalty (Cossío-Silva et al., 2019; Lee and Xue, 2020; Quoquab et al., 2020). An assumption in the literature exists that when the perceived value and experience are found in a positive perspective, this leads to a higher level of inner motivation that eventually leads to eco-tourist destination loyalty (Mirzaalian and Halpenny, 2021). The study framework is developed by considering the perceived value and experience as two factors that motivate the inner state in destination equity and destination attachment (Cifci, 2021; Kumail et al., 2021), leading to destination loyalty in the ecotourism context.

### Tourism consumption theory

Tourism consumption theory provides the theoretical base for studying the complex leisure system influenced by tourist value perception and experience associated with travel (Woodside and Dubelaar, 2002). TCT states that traveler choice, opinion, motive, and behavior are interrelated, influencing the decision-making process (Suhartanto et al., 2020a). The theory is enacted from Clawson and Knetsch (1966) five-phase model of recreation. Woodside and Dubelaar (2002) claim that leisure trip planning is a complex procedure that consists of multiple factors, such as the tourist's prior experience, prior decision-making process, and the tourist background. Woodside and Dubelaar (2002) believe that thoughts, decisions, and behavior are interdependent, and this leads to direct and indirect relationships between tourist behavioral perspectives. The prior literature has tested the theory and has shown considerable support for the study. The readers can track back the traces of earlier attempts to understand the TCT perspective on tourists, where researchers suggest that tourist decision-making prior, after, or during the travel depends on a diversified set of beliefs (Li et al., 2013; Suhartanto et al., 2021).

This makes TCT a suitable option to provide rationale between tourist perceived value and prior experience in providing the inner motivation in destination attachment and destination equity that forms the behavioral response in destination loyalty. TCT states that the prior experience of tourists results in developing destination evaluation, generating destination distinct position and bond between tourist and destination (Suhartanto et al., 2020a). In the present context, it is assumed that tourist assessment is based on the value perception and prior contact that develop attachment and destination equity, generating loyal tourists. In support of this argument, the empirical evidence (Kuo and Feng, 2013; Kim and Park, 2016; Ahn and Kwon, 2020; Suhartanto et al., 2020a) reinforces the statement that tourist value perception and experience develop the internal motives in terms of destination equity and attachment that subsequently lead to the destination loyalty.

### Perceived value

The study of consumer value perception was initiated in the early 1980s (Dodds and Monroe, 1985; Porter, 1985), and this process evolved further in the 1990s (Woodruff, 1997). The literature review shows that perceived value (PV) was introduced in the Chinese tourism sector in the late 1990s (Xia and Chen, 2015). Primarily, PV is mainly discussed in terms of utility evaluation. The utility evaluation in terms of experience, facilities, and economic value. This study considers the PV in the context of the utility evaluation approach to study tourist expectations. Thus, the study defines the PV as an evaluation of the perception of experience, facilities, and economic value compared to ordinary tourist destinations.

Perceived value is the trade-off between the perceived acquisition and the cost incurred for a particular product or service (Chua and Banerjee, 2015). Therefore, consumer intention to consume a specific product or service depends on the perceived value they have received, such as the trade-off between the perceived benefits and cost analysis. Platania et al. (2016) framed the theory of perceived value. They defined the concept of perceived value as the collective evaluation of product or service performance in comparative nature between the perceived benefits and cost incurred, while Keller and Kotler (2015) consider the value a collective evaluation of the product or service performance. In ecotourism, perceived value is a tourist evaluation of the destination and incurred costs. The prior studies show the relationship between perceived value and behavior intention (El-Adly, 2019).

### Tourist experience

The tourist experience is any event, while behavior, perception, rumor, cognition, emotion, words, gestures, or feeling is the tourism experience (Li et al., 2021). Furthermore, experience is the tourist destination interaction. During this interaction, tourists construct a unique experience (Huang and Hsu, 2009), which reflects their cognitive learning during the experience (Li et al., 2021). The construct is measured from various perspectives. Joseph and Gilmore (1998) advocate that the business market has transformed from production to service-based economies and considers the experience-defining dimension of consumer intention. Schmitt (1999) suggests studying the consumer in terms of rationale and emotions. He suggests studying consumer behavior in tourist destinations in terms of sensory, emotional, thinking, acting, and relationship perspective.

There have been limited studies to understand the tourist experience at eco-destinations. Chan and Baum (2009) defined the tourist experience in terms of hedonic, interactive, novelty, comfort, stimulation, safety, and security, creating a combination of Joseph and Gilmore's (1998) and Schmitt's (1999) studies. Wang et al. (2012) studied ecotourism in the context of aesthetic, emotional, and action perspective that is similar to the prior discussed frameworks. These empirical studies suggest that ecotourism is a blend of experiences, highlighting the importance of a singular scale to study all these perspectives. This study considers the shorter scale to study the consumer interaction with the destination (Shahijan et al., 2018).

### Destination attachment

Ren et al. (2010) found that the concept of attachment came from childhood and believed it is an outcome of dependence on parents; as individuals move forward in life, they shift this attachment to other objects, places, and environments. Lee et al. (2019) define destination attachment as the emotional bond between the place and tourist in terms of social and physical attachment. Yuksel et al. (2010) studied the tourist destination attachment and found that this concept consists of place dependence and place recognition. Prayag et al. (2018) found that tourist engagement with the place strengthens the destination attachment, leading to multiple outcomes in destination satisfaction, loyalty, and positive word of mouth. This study considers the destination attachment scale (Prayag and Ryan, 2012) due to its consistent reliability, confirmed by many other tourism studies. Based on the existing literature, Prayag and Ryan (2012) defined destination attachment as the psychological engagement with the destination, which later might generate destination-related emotional decision-making. The Psychological Continuum Model (PCM) model of Funk and James (2008) engages the destination attachment for mediating relationships to study the destination fascination and tourist loyalty. This study considers destination attachment as a mediating variable between tourist destination value and destination experience.

### Destination equity

Aaker (1991) defines brand equity in the context of assets and liabilities associated with a brand, such as a name, logo, and symbols that enhance its value to its customers. This concept of brand equity developed by Aaker (1991) and Keller (2003) helps the destination managers to develop performance measures to position the destination properly in the market to enhance the value offered by the destination to visiting tourists. Few studies provide a comprehensive model to study the concept (Kumail et al., 2021). The literature provides help to study the dimensions that lead to destination equity. For example, Kladou and Kehagias (2014) studied brand equity with four dimensions: awareness, culture, quality, and loyalty. Chi et al. (2020) explain the concept in terms of the image, perceived quality, and familiarity by studying tourism destination loyalty and travel intention. Stojanovic et al. (2017) explain the role of social media-based awareness leading to higher consideration of destination equity, generating positive or negative word of mouth enacted from the tourist perception given in terms of feedback on social platforms. This study considers destination equity as the product of perceived value and experience and the mediating role between perceived value, experience, and destination loyalty.

### Destination memory

Baddeley et al. (1999) define memory as a “systematic working alliance that helps us to learn from the past and predict the future.” Episodic memory, the long-term storage of facts, is a concern in experiences (Schwartz et al., 2011). Episodic memory is the topic of interest in the tourist experience (Larsen, 2007). Tourist experience comprises complex psychological structures focused on memory (Larsen, 2007). Scholars have given multiple definitions for the tourist experience. The destination experience is studied in subjective and individual evolution of interaction and tourist experience at the destination in terms of events, activities, and many more happenings, thus leading to long-term memories (El Haj and Miller, 2017). Larsen (2007) considers tourist experience as the mic of past and personal travel experiences which are strong enough to enter the long-term memory of tourists. Considering the context of the present study, the positive memories of the tourist experience are more relevant, and this concept is of vital importance when it comes to ecotourism (El Haj and Miller, 2017). Kim et al. (2010) state that the emergence of positive, memorable experiences leads to revisiting intention, and this tendency is of vital importance in the tourist travel-related decision-making process.

## Hypothesis development

**Figure 1:**
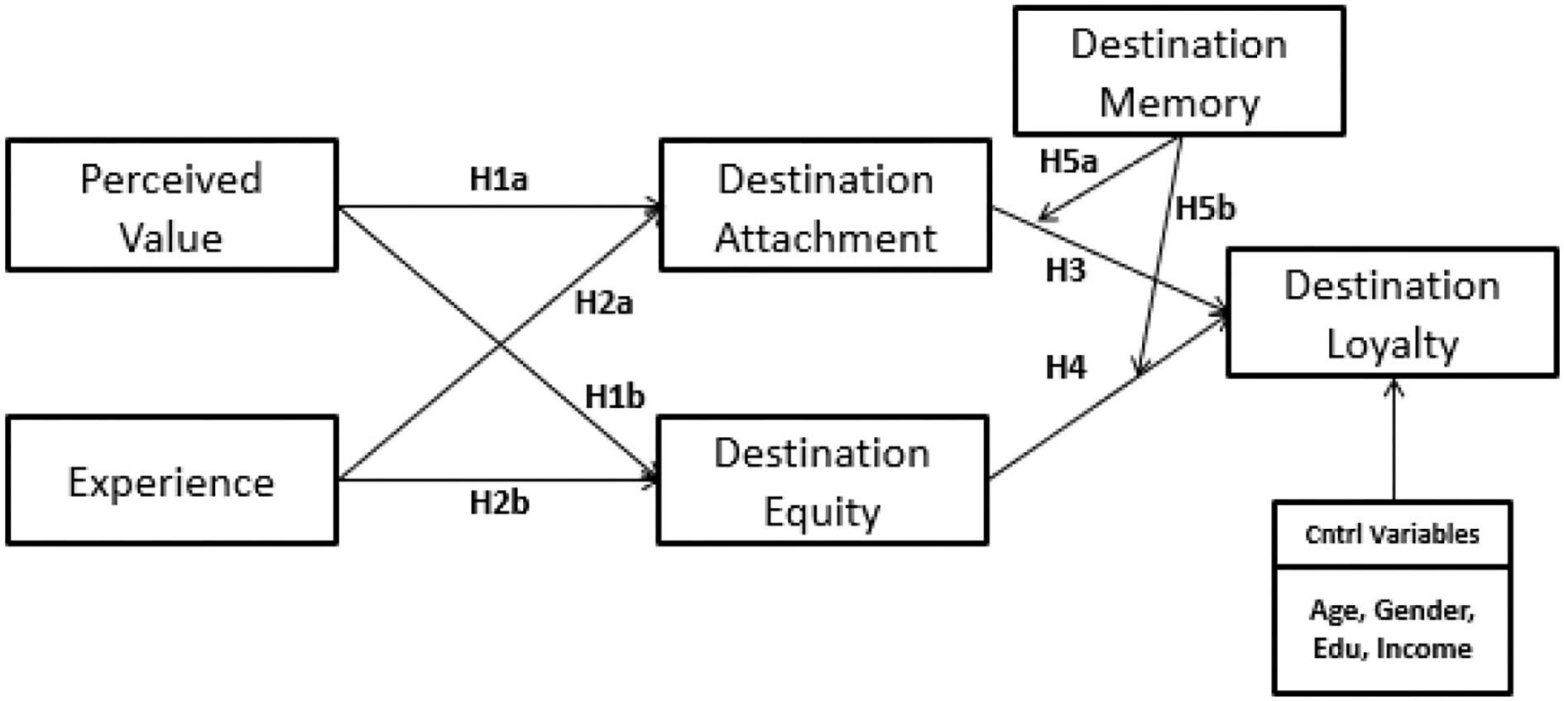
Analytical framework for the influential factors of destination loyalty.

Enacted to the existing body of literature, this study develops an analytical framework to study the variables that affect tourist destination loyalty. The graphical relationships are shown in Figure 1, and a detailed discussion is provided in this section.

### Hypothesis development

The personal evaluation of received goods and services effectiveness is developed from the perception of what is received and what was expected. The PV has received considerable attention from marketing scholars and practitioners. PV is pivotal to obtaining a competitive advantage in the market and is the only way to build and hold this advantage for a longer time. PV is the overall evaluation of the product or service developed by comparing the perceived acquisition and cost incurred (Chua and Banerjee, 2015). That is why consumer intention is usually dependent on the value received against the cost. A simple trade-off between the perceived value and perceived cost (Platania et al., 2016) coined the theory of perceived value with a customer perspective and defined the PV as an overall evaluation of offers from a destination, consequently comparing perceived benefits and cost paid. Keller and Kotler (2015) regarded the PV as a tourist evaluation of the overall effectiveness of destination offering. Alexandris et al. (2006) found that the PV influences the tourist's internal decision-making process in many ways, such as destination attachment and utilitarianism. The study states that PV is an antecedent of eco-destination attachment. Considering this perspective of discussion, the study proposes the following hypothesis:

**H1a:** Value perception increases tourist destination attachment.

Destination equity examination is a complex process in a marketing context. A marginal improvement in brand equity leads to the long-term generation of resources and tourist satisfaction (Kumail et al., 2021). Equity must be measured in terms of revenue, taxes to the government, employment, and better wages (Gartner, 2014). Any improvement in the brand is directly tied to destination equity. Ha and (Shawn) Jang (2010) found that perceived value leads to numerous behavioral outcomes, which consequently influence the internal state of mind. Ahn and Kwon's (2020) study of Malaysian green hotels states that value perception is the major factor that enhances the overall brand equity of the green hoteling concept and attracts the tourist with emotional attachment to the environment. Moreover, Chi et al. (2020) consider that the awareness of benefits associated with a destination in terms of naturalness, monitory benefits, and service quality leads to better destination equity. Similarly, Kladou and Kehagias (2014) consider brand equity an outcome of tourist perception of the value they will gain against the resources invested. Any increase in the tourist perception of the destination in terms of value results in improved visitation and spending more time at the destination. So, based on these facts, the study proposes the following hypothesis:

**H1b:** Value perception increases tourist destination equity.

The overall perception of the term experience is of vital importance in tourist-related marketing strategies, and this makes the tourism sector outperform the other industrial sectors (Shahijan et al., 2018). Moreover, eco-tourists look for up-to-date and modern services in order to enhance their traveling experience. The individual experience plays important role in the social and economic life of a tourist. Likewise, the tourist experience has gained the prime position to understand tourism. The tourist experience is a more explicit term to study the tourist destination experience in the past decade (Li et al., 2021). Tourist visit is all about the experience; they visit to experience the breadth and depth of the destination and experience its novelty (Huang and Hsu, 2009). Furthermore, experience is a mix of feelings, ideas, thoughts, and gossip taking place throughout the visit (Larsen, 2007). In the context of ecotourism, experience is defined as the “culmination of a given experience formed by tourist while they are traveling and spending time at a given destination” (Chan and Baum, 2009). Quynh et al. (2021) studied the role of experience in the context of emotions and their ability to influence the destination image, consequently affecting the destination satisfaction level. Chan and Baum (2009) consider the ecotourism experience as hedonic, interactive, novel, comforting, safe, stimulating, and vital in establishing the tourist attachment to the destination. El Haj and Miller (2017) define the tourist experience in terms of long-term memory that pushes the internal state in an emotional way and raises tourist intention to stay loyal to eco-destination tourism. This study considers that experience is a vital perspective of tourist attachment to the eco-destination. Considering this perspective, the study proposes the following hypothesis:

**H2a:** Prior experience increases tourist destination attachment.

The literature depicts that experience is a vital source of tourist satisfaction with the destination (Quynh et al., 2021). Li et al. (2021) studied the post-trip destination image and the role of destination satisfaction in forming ecotourism loyalty. Li et al. (2021) further state that the experience is of pivotal importance in framing the destination equity due to higher tourist satisfaction with eco-destination. The study found that post-experience satisfaction increases destination differentiation rather than destination equity. Moreover, Nella and Christou (2010) measured the impact of tourist experience on brand equity and market outcomes. The study highlights the important role of the tourist experience in terms of the wine industry and its role in enhancing the overall brand equity. The study elaborates on the post-visit and post-consumption experience of wine on tourist perception of brand differentiation. Gartner (2014) evaluated the post-visit destination equity of tourist resorts and found a positive link between destination equity and tourist visit that improves visitors' awareness level of tourism, eventually leading to loyalty. Based on the above-mentioned facts, this study proposes that the experience directly influences destination equity. So, the study proposes the following hypothesis:

**H2b:** Prior experience increases tourist destination equity.

Literature provides consistent evidence of a significant relationship between tourist destination attachment and their willingness to stay loyal and make revisits (Alexandris et al., 2006; Yuksel et al., 2010; Prayag and Ryan, 2012). Quynh et al. (2021) found that tourist is more likely to make revisit when they are having emotions attached, and the intention to stay loyal is higher when a strong bond exists between the tourist and destination. Prayag and Ryan (2012) state that tourist is more satisfied if they have prior attachment and affection with the destination, leading to continued loyal behavior in terms of positive word of mouth and peer recommendation. Alexandris et al.'s (2006) study explains the role of service quality factors in improving destination attachment and consequently gaining loyal tourist behavior. In terms of ecotourism, loyalty is of vital importance for the existence of business and local socio-economic activity. The existing literature has discussed ecotourism loyalty in diversified dimensions. For instance, Li et al. (2021) found that post-trip satisfaction develops a sense of attachment, leading to eco-tourist destination loyalty. Similarly, Xia and Chen (2015) state that tourist pro-environmental behavior leads to emotional attachment to eco-destination. Considering these facts, the study proposes the following hypothesis:

**H3:** Destination attachment enhances tourism destination loyalty.

Elements of destination equity are developed from the tourist perspective. Destination equity has a significant relationship with visiting tourist intentions (Kladou and Kehagias, 2014). Destination equity helps the tourist to differentiate between different destinations based on their personal perception of value and experiences during the visit (Gartner, 2014). In ecotourism, the tourist perception of destination equity plays a vital role in keeping the tourist loyal to the destination. Malik et al.'s (2021) study found that green attributes of destination matter for the tourist, and tourists with favorable attitudes will show an intent to stay loyal due to higher destination brand equity. Similarly, Nella and Christou (2010) studied the role of real-time experience in product manufacturing and suggested that tasting experience can contribute strongly to destination equity. Stojanovic et al. (2017) found the effective role of social media in developing higher destination equity. The study found that how tourist discusses the experience of eco-destination on social media leads to better awareness of peers, thus leading to destination equity. So, the study proposes the following hypothesis:

**H4:** Destination equity enhances tourism destination loyalty.

Joseph and Gilmore (1998) state that tourist experience gets stored in long-term memory. The experience that tourist has gained during interaction with the destination push them to recommend, develop positive word of mouth, and revisit a specific destination (Malik et al., 2021). Memory is thought to be the prime indicator of the tourist travel decision-making process, and destinations with positive experiences and emotions are more likely to be remembered and considered for the next holidays (El Haj and Miller, 2017; Kim, 2020). Not only do the positive experiences and memory contribute to the final decision, but also the negative experiences leading to negative thought process is also remembered for a long time (Kim, 2020). Considering this pivotal role of tourist memory of destination motivates to consider the moderating role between the destination attachment, destination equity, and destination loyalty. The study proposes the following hypothesis:

**H5a:** Destination memory improves the association between destination attachment and destination loyalty.

**H5b:** Destination memory improves destination equity and destination loyalty.

Recent studies show that destination attachment and destination equity play a mediating role between the behavioral antecedents and tourist intentions (Prayag and Ryan, 2012; Chi et al., 2020). Nasyat et al. (2020) used the destination attachment between the destination attractiveness and visit intentions. This study considers destination attachment as a mediating construct between perceived value, experience, and destination loyalty. Similarly, Liu et al. (2015) studied the mediating role of brand equity between the consumer intention to visit the museum and allied behavioral outcomes. This study considers the mediating role of brand equity between the perceived value, experience, and destination loyalty. So, the study proposes the final two hypotheses as follows:

**H6a**. Destination attachment mediates the relationship between perceived value and destination loyalty.

**H6b**. Destination attachment mediates the relationship between perceived benefits and destination loyalty.

**H6c**. Destination equity mediates the relationship between perceived value and destination loyalty.

**H6d**. Destination equity mediates the relationship between perceived benefits and destination loyalty.

## Methodology

The prime objective of the present study is to evaluate the tourist intention to stay loyal to eco-destinations for the tourist decision-making process with a moderating role of destination memory and a mediating role of destination attachment and destination equity. The study is quantitative and descriptive in nature. Furthermore, the study considers the deductive approach, as the study is enacted on the existing body of literature. However, the study follows the cross-sectional approach to gather data from respondents. A questionnaire-based survey technique is adapted to attain the respondent response through online means.

### Measurements

All the construct measurements are adapted from the existing body of literature that helps us to ensure the construct's reliability and validity in the current context. The construct items for perceived value are adapted from the study of Suhartanto et al. (2020a). The construct items for the tourist experience of a destination are adapted from the study of Shahijan et al. (2018). Furthermore, the construct items for the mediating variables of destination attachment and destination equity are adapted from the study of Reitsamer et al. (2016) and Baalbaki and Guzmán (2016). Moreover, the construct item for the eco-tourist destination loyalty is adapted from the study of Wu (2016), whereas, the scale items for the moderating role of destination memory are adapted from the study of Ali et al. (2014). The respondent's response is obtained with the help of a Likert seven-point scale, with “7” indicating strongly disagree and “1” indicating strongly agree.

### Population and sample

The population sample of this study comprises tourists who have prior experience in visiting ecotourism destinations in China. To ensure this, the questionnaire had an opening statement asking the respondents whether they have visited an eco-tourist destination or not. The data are collected from the major cities of the country, such as Beijing, Shanghai, Guangzhou, Nanjing, Tianjin, Wuhan, and Hefei. Before the data collection campaign, the pretesting was done and the questionnaire was handed over to 15 postgraduate experts. The minor adjustments were made according to the recommendations from experts. Considering the COVID-19 protocols, the data were collected through the online circulation of questionnaires (www.wjx.cn). The survey respondents were assured that the given information would be kept secret and used for research purposes only. Through wjx, 58 responses were attained, and all were found to be fit for further processing. Table 1 presents the demographic details of the survey respondents.

**Table 1:** The demographic detail of survey respondents.

**Demographic analysis**			
	**Item**	**Total**	**%**
Gender	Male	181	50.6
	Female	177	49.4
Age	Below 20	36	10
	21–25	125	34.91
	26–30	110	30.72
	31–35	80	22.34
	36 and above	7	2
Occupation	Students	95	26.53
	Professional	131	36.59
	Businessman	100	27.93
	Other	32	8.93
Income (RMB)	Below 50 thousand	131	36.59
	51–99 thousand	140	39.10
	100–199	29	08.10
	200+	58	16.20
Education	Graduation	152	42.45
	Masters	140	39.10
	Mphil/PhD	66	18.43

*n = 358*.

**Table 2:** Construct items, factor loading, AVE, Cronbach's alpha, CR, *R*^2^, and *Q*^2^ values are presented.

**Construct items**		**VIF**	**Loading**	**α**	**AVE**	**CR**	**R^2^**	**Q^2^**
**Perceived value**								
PV1	The Eco destination has good value for money.	1.448	0.831	0811	0.635	0.874		
PV2	The eco-destination fee is reasonable.	1.663	0.778					
PV3	The eco-destination makes me accepted by others.	1.533	0.770					
PV4	The eco-destination makes me happy.	1.771	0.808					
**Experience**								
EX1	Eco destination experience was stimulating	1.881	0.779	0.842	0.612	0.887		
EX2	Eco destination experience was exciting	1.883	0.776					
EX3	Eco destination experience was enjoyable	1.642	0.790					
EX4	Eco destination experience was interesting	1.813	0.845					
**Destination attachment**								
DA1	Eco destination is the best place for what I like to do on holidays	1.773	0.684	0.704	0.528	0.816	0.518	0.256
DA2	I am very attached to Eco destination	2.059	0.746					
DA3	Holidaying in Eco destination means a lot to me.	2.991	0.652					
DA4	No other place can provide the same holiday experience as Eco destination.	1.987	0.814					
**Destination equity**								
DE1	Eco destination is an environmentally safe destination.	1.169	0.698	0.729	0.509	0.774	0.479	0.217
DE2	Eco destination is an environmentally responsible destination.	1.431	0.840					
DE3	Eco destination is a sustainable destination.	1.386	0.860					
DE4	Eco destination is a healthy destination.	1.602	0.649					
**Destination loyalty**								
DL1	I would recommend others to visit Eco destination.	1.987	0.789	0.758	0.674	0.861	0.569	0.374
DL2	I will visit Eco destination in the future	2.159	0.841					
DL3	Eco destination is my first choice among destinations	1.194	0.831					
**Destination memory**								
DM1	I have beautiful memories of this visit to Eco destination.	2.159	0.839	0.813	0.729	0.889		
DM2	I won't forget my experience visiting an Eco destination	2.151	0.822					
DM3	I will remember many positive things about Eco's destination visit.	1.500	0.677					

Table 1 presents a summary of the respondent profile. The table shows that out of 358 respondents, 181 are male respondents (50.6%) and 177 are female respondents (49.4%). Furthermore, the respondents are segmented on the basis of age group as follows: 20, 21–25, 26–30, 31–35, and 36 and above years with a percentage of 10, 34.91, 30.72, 22.34, and 2%, respectively. Similarly, respondents are divided into four occupational categories: students, professionals, businessmen, and others with percentages of 53, 36.59, 27.93, and 8.93%, respectively. On the basis of income, 36.59% earn below 50,000 RMB, 39.10% earn between 51,000 and 99,000 RMB, 8.10% earn between 100,000 and 199,000 RMB, and only 16.20% earn more than 200,000 RMB a year. Finally, respondents are classified based on their educational qualification as graduation, master's, and MPhil/Ph.D. with a percentage of 42.45, 39.10, and 18.43%, respectively. The rest of the demographic details are given in Table 1.

## Results

To study the structural model of the study, structural equation modeling (SEM) is implied through the partial least method (PLS). The SmartPLS is the second-generation software that is used to run the measurement model and structural model simultaneously and estimate the regression and component factors together (Hair et al., 2010). The SmartPLS-based SEM is preferred over CB-SEM, as the software is empowered to run regression analyses along with the ability to run complex models having multiple variables (Hair et al., 2010). In SEM, the study model is tested through measures known as the measurement model (reliability, convergent validity, and discriminant validity), and the second procedure checks the interrelationship between the variables known as the structural model (Hair et al., 2010).

The study engages the SmartPLS 3.2.8 version for data analysis.

### Measurement model

Social science studies have to ensure the construct's reliability and validity. The current study adopts the set of necessary measures, such as internal consistency, convergent validity, and discriminant validity, to ensure this. First, the study measures Cronbach's alpha (α), factor loading, composite reliability (CR), and average variance extracted (AVE). The results show that all the values are above the threshold levels of factor loading (0.7), CR (0.5), and α (0.6) (Hair et al., 2010; Hair, 2016). The results of Cronbach's alpha, factor loading, composite reliability and average variance extracted are given in Table 1. Furthermore, the study checks the discriminant validity to verify the internal variance between the convergent validity (Henseler et al., 2014). The statistical results show satisfactory findings as given in Table 3. The results exhibit valid discriminant validity.

**Table 3:** The discriminant validity.

	**DA**	**DE**	**DL**	**DM**	**EX**	**PV**
Destination attachment (DA)	**0.727**					
Destination equity (DE)	0.807	**0.686**				
Destination loyalty (DL)	0.662	0.684	**0.821**			
Destination memory (DM)	0.622	0.715	0.672	**0.854**		
Experience (EX)	0.656	0.571	0.634	0.565	**0.782**	
Perceived value (PV)	0.665	0.676	0.748	0.714	0.683	**0.797**

*HTMT ratio in bold less than 1 is acceptable criterion (Henseler et al., 2014)*.

#### HTMT criterion

The third way used in this study to check the discriminant validity is the heterotrait–monotrait ratio of correlations (HTMT). The threshold value is 0.9 to ensure suitable discriminant validity (Henseler et al., 2014). The outcome depicts that all the values are below the cut-off value.

#### Collinearity statistics

Collinearity posits that a predictor variable can predict another variable in multiple regression models. This happens due to the correlation measured through the variance inflation factors (VIF). The cut-off value of VIF ranges from 3.3. to 10 (Schlittgen et al., 2016). The VIF value for this study ranges from 1.014 to 2.991, which is within the cut-off value. So, we can claim that this study does not have a multicollinearity issue.

### Common method variance

Common method variance is a vital concern when data are collected from a single source. This study performs HTMT and VIF tests to verify its existence. Besides these tests, the study performed the Harman single test via exploratory factor analysis through SPSS software. This process categorizes the all-construct items into six subgroups. The first factor explains only 23.80% of variance which is far less than the maximum point value of 40%. Furthermore, the study compares and evaluates the six-factor research model with the help of a single-factor and two-factor model with the SEM, where each factor has three variables and informants that deliver the data to these variables. The six-factor model results (*X*^2^ = 1,255.51, *df* = 768) in a better fit then the single-factor model (*X*^2^ = 4,821.35, *df* = 265) and the two-factor model (*X*^2^ = 8,401, *df* = 813). Furthermore, the study uses the marker variable, the one that is not related to this study or any of the variables of the current study (Williams et al., 2010). The study outcome shows that the interrelationships between the latent variables are not influenced by the CMV. So, this study does not have any issues with CMV (Siemsen et al., 2009).

### Structural model

The structural model is used to check the paths between the constructs and their allied influence. This study has a theoretical framework, as given in Figure 1, as the two-step model assessment was done through SmartPLS. Structural equational modeling is done through the collinearity and path significance coefficient.

#### Structural model and hypotheses testing

The study uses a structural path coefficient to measure the SEM with the help of SmartPLS. The path significance is cross-verified through the bootstrapping technique. Furthermore, we measure the R-square value along with the path analysis. The SmartPLS can predict around 5,000 sample sizes simultaneously. The coefficient of confidential internal is measured at 95% or t > 1.96 with the help of two-tailed tests (Schlittgen et al., 2016).

**Figure 2:**
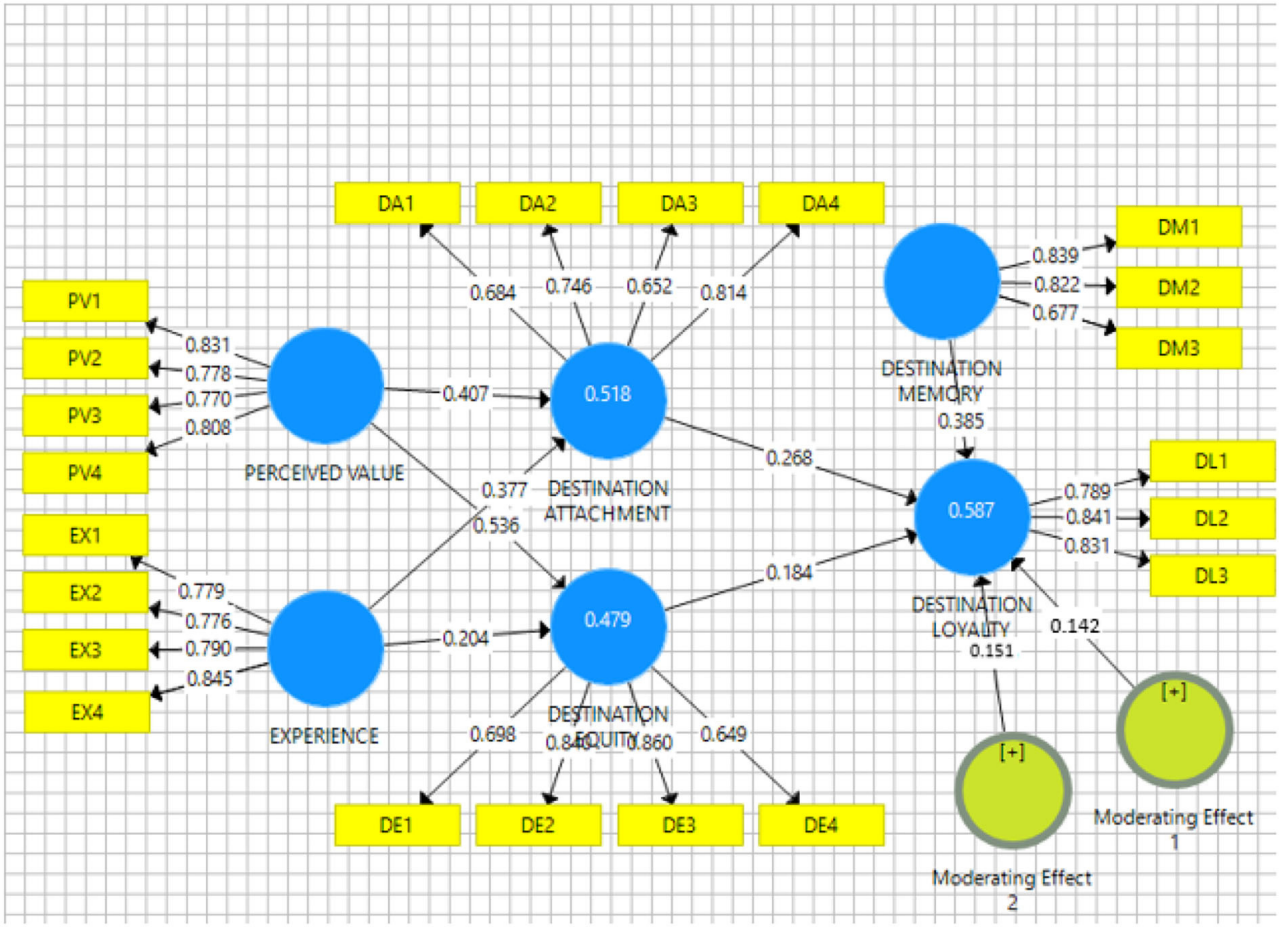
The path coefficients of the structural model are presented.

The coefficient of determination (*R*^2^) represents the proportion of variation in the dependent variable. The value of *R*^2^ > 0.2 is a suitable and reliable outcome (Henseler et al., 2009). The *R*^2^ values for the current study are above the cut-off value of 0.2 (Urbach and Ahlemann, 2010). For destination attachment (*R*^2^ = 0.518), results show that 51.8% of the variation in destination attachment is caused by the perceived value and experience. A value of *R*^2^ = 0.479 for destination equity means that 47.9% of the variation in destination equity is caused by the perceived value and experience. A value of *R*^2^ = 0.569 for destination loyalty means that 56.9% of the variation in destination loyalty is caused by the tourist destination attachment and destination equity. Figure 2 presents the *R*^2^ values of the current study.

The outcome shows that perceived value contributes positively to destination attachment (β = 0.407, *p* < 0.001) and destination equity (β = 0.0.536, <0.001). Hence, H1a and H1b are supported. Moreover, experience also contributes positively to the destination attachment (β = 0.377, *p* < 0.001) and destination equity (β = 0.204, *p* < 0.001). So, H2a and H2b are also supported. Furthermore, the results show that destination attachment positively contributes to eco-tourist destination loyalty (β = 0.268, *p* < 0.001); therefore, H3 is supported. In addition, destination equity also contributes positively to eco-tourist destination loyalty (β = 0.184, *p* < 0.001). Hence, H4 is also supported. Table 4 presents the structural path analysis results along with the appropriate significance level.

**Table 4:** Summary of structural path model results.

**S.No**.	**Hyp**.	**Relation**	**Sample mean (M)**	**Standard deviation (STDEV)**	***T*-Test (|O/STDEV|)**	***P***	**Outcome**
1	H1a	PV-> DA	0.407	0.046	8.823	0.001	Supported
2	H1b	PV-> DE	0.536	0.040	13.439	0.001	Supported
3	H2a	EX->DA	0.377	0.042	9.075	0.001	Supported
4	H2b	EX->DE	0.204	0.043	4.696	0.001	Supported
5	H3	DA->DL	0.268	0.053	4.921	0.001	Supported
6	H4	DE->DL	0.184	0.061	2.881	0.001	Supported

*PV, perceived value; EX, experience; DA, destination attachment; DE, destination equity; DL, destination loyalty; DM, destination memory, Hyp, hypothesis*.

#### Blindfolding

The blindfolding procedure measures the relevance between the exogenous variables to predict the structure's performance. It is just a reuse of the said procedure (Mikalef et al., 2017). Blindfolding is a mix of function fitting and cross-validation. This technique measures the constructability to predict the relevance by observing the change in criterion estimates (*Q*^2^) (Hair et al., 2012). The results of Stone-Geisser's blindfolding show that destination attachment (*Q*^2^= 0.256), destination equity (*Q*^2^= 0.217), and ecotourism destination loyalty (*Q*^2^= 0.374) are acceptable, and all the constructs have suitable predictive relevance.

### Moderation

**Figure 3:**
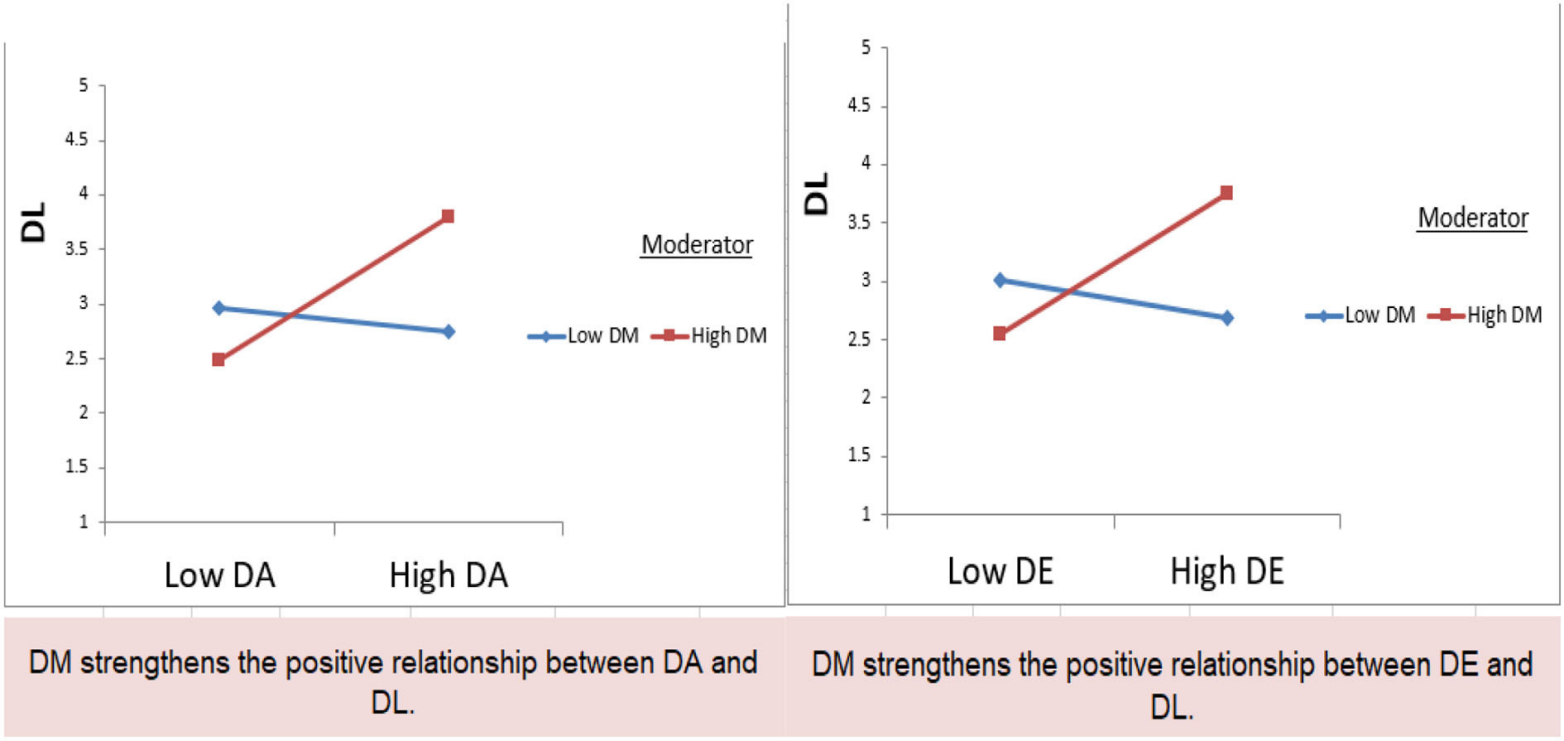
The graphical interpretation of moderating relationship is presented.

The hypotheses H5a and H5b are about the moderating role of destination memories. The results show that destination memory moderates the relationship between destination attachment and destination loyalty. Rather, destination loyalty strengthens the positive relationship between destination attachment and destination loyalty (β = 0.142, *p* < 0.05). Furthermore, destination memory also moderates the relationship between destination equity and destination loyalty. The results exhibit that destination memory strengthens the positive relationship between destination equity and destination loyalty. So, H5a and H5b are supported. Furthermore, Figure 3 presents the graphical interpretation of the moderating relationship.

### Mediation

This study shows the mediating role of destination attachment and destination equity between the perceived value, experience, and destination loyalty. The study offers four mediating hypotheses (H6a, H6b, H6c, and H6d). The mediation analysis is done with the help of variance accounted for (VAF). VAF is done by dividing the indirect effect by the total effect and multiplying it by 100 to measure the mediation effect (Hair et al., 2013). The total effect is measured by adding the direct and indirect path coefficients with the mediator and the addition of the mediator. We estimate the partial, no, or full mediation based on the criterion of a previous study (Hair et al., 2013). The mediation relationship is considered partially mediating between 20% and 80%. When average is more than 80% its fully mediating relationship (Hair et al., 2013). The mediating outcome of the study exhibits that all the mediating hypotheses partially mediate. This further enhances the credibility of our outcome. The mediation output is given in Table 5.

**Table 5:** Mediation analysis.

**Mediation outcome of perceived value and experience**							
**Hyp**.	**Regression path**	**Direct effect**	**Indirect effect**	**Total effect**	**Variance accounted for (VAF)**	**Mediation results**	**Decision**
H6a	PV->DA->DL	0.207	0.109	0.407	0.109/0.407*100 =26.78%	Partial mediation	Supported
H6b	PV->DE->DL	0.207	0.121	0.536	0.121/0.536*100 =22.57%	Partial mediation	Supported
H6c	EX->DA->DL	0.138	0.097	0.377	0.097/0.377*100 =25.72%	Partial mediation	Supported
H6d	EX->DE->DL	0.138	0.101	0.204	0.124/0.264*100 =49.50%	Partial mediation	Supported

*PV, perceived value; EX, experience; DA, destination attachment; DE, destination equity; DL, destination loyalty; DM, destination memory; Hyp, hypothesis*.

## Discussion

The contribution of tourist loyalty to the success, progress, and prosperity of an eco-tourist destination is well-established in the literature. Therefore, investigating the destination loyalty antecedents and consequent behavioral response is of vital importance for academics and managers (Ali et al., 2014). A gap exists in the literature regarding the behavioral antecedents of the eco-tourist and their intention to stay loyal to ecotourism destinations (Quoquab et al., 2020). Although the literature provides ample evidence of ecotourism visitation intention, the concept of tourist loyalty to eco-destination is embedded in the tourism industry, which makes it essential to test the concept with different frameworks (Ali et al., 2014). The objective of the current study is to evaluate the influence of destination attachment and destination equity ability in developing destination loyalty in light of perceived value and experience. Furthermore, the study evaluates the mediating role of destination attachment and destination equity between the perceived value, experience, and destination loyalty. Moreover, the study offers the moderating role of destination memories between destination attachment, destination equity, and destination loyalty. To achieve these objectives, the study enacted a model from the existing literature in light of TCT to study tourist behavioral intentions.

The statistical results found a positive and significant relationship between perceived value, destination attachment, and destination equity, which supports H1a and H1b. The results are consistent with the previous findings of Ahn and Kwon (2020). The study of Ahn and Kwon (2020) found that green hotels' value perception positively contributes to the revisit intention of guests. Furthermore, they claim that perceived value contributes to the positive and negative emotions that ultimately develop the revisit intention. Moreover, perceived value makes a significant contribution to the dependents, as the same time factor loading (0.831) reveals that value for money is the prominent factor in shaping positive destination attachment and destination equity in comparison to the other construct items.

However, this study outcome shows that perceived value contributes to destination attachment and destination equity that lead to the consequent behavioral response in terms of loyalty to the eco-tourist destination. This study outcome highlights the important role of perceived value and its ability to influence the internal mental state of tourists. This outcome is further validated with the mediation role of destination attachment and destination equity between the perceived value and tourist destination loyalty, thus supporting H6a and H6b. The mediation findings are indirectly in line with the prior studies of Nasyat et al. (2020) who found the mediating role of destination attachment between user satisfaction and destination loyalty. Furthermore, destination equity (Kumail et al., 2021) mediates the relationship between brand authenticity and destination visit intentions. This alignment with the existing literature connects the current study with prior literature and validates the findings of the study.

In the same manner, the tourist experience positively contributes to developing destination attachment and destination equity, supporting H2a and H2b. These findings are in line with the prior literature. The study of Cifci (2021) found that memorable trip memories lead to greater post-visit satisfaction and destination attachment. Similarly, the study of Quadri-Felitti and Fiore (2013) shows the change of mind and consumption intention of wine tourists after experiencing the production facilities. Kladou and Kehagias (2014) highlight the vital contribution of brand equity in developing the brand image and brand attachment. Furthermore, in terms of eco-destination, a previous study (Gartner, 2014) found a strong contribution of brand equity in developing tourist intentions to visit the eco-destinations. This extends the destination equity literature and finds that the user experience of prior visitation to eco-destination is crucial in the decision-making process. In this context, this study's findings show that prior experience of tourists to eco-destination contributes positively to destination attachment and destination equity. The findings of this study are cross-checked through the mediating role of destination attachment and destination equity between the experience and destination loyalty to ecotourism destination, thus supporting H6c and H6d. Furthermore, the dominant factor in tourist experience is the tourist interest in the destination with a prominent factor loading value of 0.845. This outcome will help the policy-makers to make the tourist eco-experience interesting in terms of learning, excitement, and happiness.

Further, the tourist attachment to the destination and positive perception of the brand equity lead to ecotourism destination loyalty, thus supporting H3 and H4. Destination attachment makes a positive and significant contribution to destination loyalty in the context of ecotourism, and these findings are directly in line with the previous literature (Reitsamer et al., 2016). The factor loading reveals that the prominent factor that contributes to the destination attachment and its consequent result in terms of loyalty is the holiday experience (0.814) at the destination. These findings are in line with the prior literature that exhibits a strong relationship between tourist holiday experience and revisit intention (Suhartanto et al., 2021).

Similarly, the perception of destination equity makes a positive and significant contribution as observed in a prior study (Gartner, 2014). The construct has four adopted items from the prior literature, and the sustainability of eco-destination (0.860) is the prime contributor. The prior literature shows that brand equity or destination equity is due to higher social recognition, emotions, or cognitive attachment (Dwivedi et al., 2019). The present study extends the equity literature in terms of ecotourism and suggests that policy-makers must highlight the sustainable conception of ecotourism. This study extends the destination attachment and equity findings to the ecotourism context.

In the end, the moderating role of memories of prior interaction with the tourist destination is important to motivate the tourist to stay loyal to a specific destination. The destination memory moderates between the destination attachment and destination equity. This extends the existing body of literature in terms of destination memory and its important role in the decision-making process. These findings support the argument of El Haj and Miller (2017), who find that memory and social cognition have a direct relationship. The tourist memory plays an important role in processing the information, remembering, and using the information to make future decisions. This finding is in line with the current study, which shows the vital role of destination memory and its ability to influence tourist decision-making in terms of eco-destination loyalty. However, among the construct items, the beautiful experience (0.876) of destination is the leading factor, closely followed by the experience (0.869).

### Implications

Just like other studies, this study has some vital theoretical and practical implications for both academics and practitioners. The following section presents the theoretical and practical implications separately.

#### Theoretical implications

First, the present study makes an important contribution to the theory by introducing a robust framework to examine tourist loyalty to the eco-destination. Furthermore, this framework is embedded in the tourist consumption theory. This study extends the concept of TCT into the eco-tourist destination loyalty literature. The theory claims that tourist motivation for a destination is influenced by a diversified set of factors (McIntyre, 2007; Suhartanto et al., 2020b). This study finds that destination attachment and destination equity are prime antecedents of tourist loyalty to eco-destinations in light of perceived value and experience.

Second, the empirical framework of the study presents the important relationships in terms of mediating the role of destination attachment and destination equity. The study results show that destination attachment and destination equity mediate the relationship between the perceived value, experience, and ecotourism destination loyalty. This embeds the current study with the existing body of literature (Suhartanto et al., 2020b; Cifci, 2021).

Third, this study extends the existing body of literature by introducing the moderating role of destination memory. The results exhibit that destination memory strengthens the already existing relationship between destination attachment, destination equity, and ecotourism destination loyalty. Memory is the vital ingredient in revisiting and staying loyal to the eco-destination (El Haj and Miller, 2017).

Fourth, this study is vital considering the Chinese data, as China is one of the leading countries in terms of natural resorts covering 14.7% of the country's land or 147 million hectares (Daxueconsulting, 2016). The present study will act as a motivational tool for further exploration to understand the Chinese tourist loyalty toward eco-destinations.

#### Managerial implications

Despite the theoretical implications, this study has some suggestions for practicing managers and business owners working in the tourist industry, particularly the ecotourism destination business development sector.

First, the tourist perception of value in terms of monetary, social, and service perspective is an important factor in developing the emotions leading to destination attachment and enhancing destination equity. The ecotourism development managers must focus on enhancing the value perception of eco-destination to gain vital tourist loyalty to keep the sector financially sound.

Second, tourist experience in terms of prior interaction also helps the tourists to make future considerations of visiting a particular type of destination. The public and private institutions involved in ecotourism management must work together in a quest to make the eco-destination experience socially and financially acceptable to the tourists.

Third, destination attachment and destination equity play a decisive role in framing ecotourism destination loyalty. In this context, business managers must focus on enhancing the destination followers by offering improved services, sightseeing, nighttime activities, and other facilities to improve the destination experience and value perception to gain a larger and stronger loyal tourist base.

Forth, destination memories make a vital contribution to enhancing the relationship strength between destination attachment and destination equity. Furthermore, memories that are much recognized and appreciated by society are remembered for a longer period of time and make better contributions to future decision-making processes (El Haj and Miller, 2017). The business managers can enhance the social coverage of tourist participation in ecotourism by sharing the key moments and destinations on social media during the visit to eco-destination.

## Conclusion

The study highlights the important antecedents of destination attachment and destination equity in light of the perceived value and tourist experience, eventually improving tourist loyalty toward the eco-destinations. Furthermore, the study offers the mediating role of destination equity and destination attachment between the perceived value, experience, and eco-destination loyalty, along with the moderating role of destination memory. The study uses SmartPLS to support the empirical findings. Moreover, the study makes theoretical and practical implications for practitioners and theory.

## Limitations

This study is quantitative research in nature. This study is limited to the possibilities of generalization of the findings to the ecotourism destinations of China only. So, the scope of the study is to be augmented with further datasets of different regions and destinations to confirm the findings of the framework. Furthermore, the study lacks the qualitative approach, to obtain an in-depth understanding of eco-destination loyalty. Hence, it is suggested that more qualitative research intervention should be done with the help of focus group discussions, interviews, observations, and content analysis. Moreover, future studies can opt for more psychological characteristics, such as emotional involvement with environmental stability, to study the consumer motivation toward eco-destination loyalty.

Ethical declaration: The ethical clearance was granted by the Tourism College, Inner Mongolia Normal University, Hohhot010022, Inner Mongolia, China, and informed consent was obtained from the participants prior to the study.

## Data availability statement

The raw data supporting the conclusions of this article will be made available by the authors, without undue reservation.

## Ethics statement

The studies involving human participants were reviewed and approved by Ethical clearance granted by the Tourism College, Inner Mongolia Normal University, Hohhot010022, Inner Mongolia, China. The patients/participants provided their written informed consent to participate in this study.

## Author contributions

MM is responsible to have the initial draft, data collection, and conceptualization. MN helped in data collection and analysis. MS designed research, revised draft, and analysis. All authors contributed to the article and approved the submitted version.

## Conflict of interest

The authors declare that the research was conducted in the absence of any commercial or financial relationships that could be construed as a potential conflict of interest.

## Publisher's note

All claims expressed in this article are solely those of the authors and do not necessarily represent those of their affiliated organizations, or those of the publisher, the editors and the reviewers. Any product that may be evaluated in this article, or claim that may be made by its manufacturer, is not guaranteed or endorsed by the publisher.
